# The effects of a Self-Alert Training (SAT) program in adults with ADHD

**DOI:** 10.3389/fnhum.2015.00045

**Published:** 2015-02-10

**Authors:** Simona Salomone, Grainne R. Fleming, Jacqueline M. Shanahan, Marco Castorina, Jessica Bramham, Redmond G. O’Connell, Ian H. Robertson

**Affiliations:** ^1^Trinity College Institute of Neuroscience, Lloyd Institute, Trinity College DublinDublin, Ireland; ^2^St. Patrick’s University HospitalDublin, Ireland; ^3^School of Psychology, University College DublinDublin, Ireland; ^4^School of Psychology, Trinity College DublinDublin, Ireland

**Keywords:** ADHD, Self-Alert Training, CAARS, biofeedback, everyday life, attention

## Abstract

Attention-Deficit/Hyperactivity Disorder (ADHD), a neuropsychiatric condition characterized by attention and impulsivity problems, is one of the most common behavioral disorders. The first line of treatment for ADHD is psychostimulant medication, but this has limited effectiveness, particularly in adults, and is often associated with adverse side-effects. Thus, it is imperative that new non-pharmaceutical approaches to treatment are developed. This study aims to evaluate the impact of a non-pharmacological Self-Alert Training (SAT) intervention on ADHD symptom prevalence, psychological and cognitive functioning, and on everyday functional impairment in adults with ADHD. Fifty-one adult participants with a current diagnosis of ADHD were randomized to either SAT or a Control Training (CT) program. They were assessed at baseline, immediately following the 5-week training period, and after 3-months using ADHD symptoms scales, as well as a series of neuropsychological tests and psychological questionnaires. Subjective ratings of everyday life attention and memory problems were also collected. The SAT group showed significant improvements in ADHD inattentive and impulsive symptoms, depressive symptoms and in self-efficacy ratings compared to the CT group at both post-training and at the 3-month assessment. Pre-post improvements in SAT participants on untrained cognitive tasks measuring selective attention and executive functions were also observed. Finally, the SAT group reported improved subjective ratings of everyday life attention at both assessment points. This pattern of results suggests that SAT may be beneficial in treating ADHD symptoms as well as psychological and cognitive impairments in adult ADHD. A large-scale randomized controlled trial (RCT) is needed.

## Introduction

Attention-Deficit/Hyperactivity Disorder (ADHD) is a neuropsychiatric condition characterized by difficulties with attention, impulsivity and overactivity. ADHD is primarily a behavioral disorder but individuals also exhibit impairments in a number of cognitive domains, particularly in higher-level executive functions such as inhibition and attention (Castellanos and Tannock, 2002; Barkley, 2006). Although it has been thought of as a disorder most commonly present in childhood, it is now known that ADHD can persist into adulthood in some cases (Faraone et al., 2006; Kessler et al., 2006; Miller et al., 2007). Research suggests that adults with ADHD have lower economic status, higher levels of unemployment (Sobanski et al., 2007), and more frequent interactions with the criminal justice system (Fischer et al., 2007). Given the high cost to society of ADHD treatments (Matza et al., 2005), with co-morbid disorders and other associated problems such as higher criminality rates (Fischer et al., 2007), it is imperative that effective interventions for these individuals are developed.

The most common treatment for ADHD is the administration of psychostimulants which, among other effects, modify dopaminergic and noradrenergic activity in the brain (Madras et al., 2005). However, used alone, pharmacological approaches to treatment have a number of disadvantages, including side effects, high associated costs for long-term prescriptions and limited effectiveness, especially in adults (Asherson, 2005; Biederman, 2005). Behavioral interventions, such as Cognitive Behavioral Therapy (CBT), based on operant conditioning principles have also been shown to reduce primary and secondary behavioral symptoms of ADHD (Safren, 2006; Solanto et al., 2008). Furthermore, previous research on psychosocial interventions and psychoeducation in adults with ADHD have shown that these behavioral treatments can improve adult ADHD symptoms and reduce associated psychiatric symptoms, such as anxiety and depression (Stevenson et al., 2003; Vidal et al., 2013). However these positive effects can be short-term and these behavioral treatments can be difficult to implement. This may explain why, despite long-term treatments, behavioral and neuropsychological abnormalities associated with ADHD can persist into adulthood (Woods et al., 2002). This has prompted a move toward identifying novel treatment strategies that can address the underlying cognitive and behavioral roots of the disorder.

In an effort to meet this need, O’Connell et al. (2008) developed a novel cognitive training strategy that targets deficits in sustained attention. Impaired sustained attention is a hallmark symptom of ADHD (Woods et al., 2002; Castellanos et al., 2005; Mcavinue et al., 2012). The available evidence from fMRI, PET and pharmacological studies indicates that sustained attention is achieved through a primarily right lateralized, multimodal cortical network that includes the anterior cingulate gyrus, the right dorsolateral prefrontal cortex and the inferior parietal lobule with prominent reciprocal connections to the thalamus and noradrenergic brainstem targets (Peterson and Posner, 2012). The cortical sustained attention network also modulates firing rates in subcortical arousal structures thus helping to maintain the state of alertness during current tasks (Foucher et al., 2004). Studies using different variants of the Continuous Performance Tasks (CPT; Losier et al., 1996; Woods et al., 2002) have consistently found sustained attention deficits in adults with ADHD. Furthermore, several studies have demonstrated sustained attention deficits in people with ADHD in the Sustained Attention to Response Task (SART; O’Connell et al., 2006; Mcavinue et al., 2012) that has been shown to activate right fronto-parietal sustained attention networks (Manly et al., 2003).

Top-down influences on arousal have been explored in studies using biofeedback techniques (Critchley et al., 2002; Lubar, 2003). During biofeedback participants receive real-time visual or auditory information conveying the current level of an otherwise covert biomarker and learn to exert volitional control over that process (Critchley et al., 2002; Nagai et al., 2004). One arousal biomarker that can be modulated during biofeedback interventions is electrodermal activity (EDA) that is recorded as changes in electrical conductance in the skin known as the Skin Conductance Response (SCR; Dawson et al., 2000). The autonomic system is subject to descending cortical and subcortical influences on hypothalamic and brainstem mechanisms and there is evidence that volitional modulation of SCR during biofeedback activates many of the same frontal control regions that have been implicated in top-down sustained attention (Critchley et al., 2002). This provides a basis for hypothesizing that training participants to modulate their SCRs should lead to improvements in sustained attention and associated impulsive behavior.

O’Connell et al. (2008) have investigated this hypothesis by examining an endogenous technique called Self-Alert Training (SAT) which is based on cognitive rehabilitation principles and it seeks to capitalize on the known relationship between sustained attention and arousal. The goal of SAT is to teach participants to transiently increase their arousal at regular intervals in order to offset the periodic decreases in endogenous control that determine momentary lapses of attention. The behavioral strategies involved in SAT arise from an earlier intervention developed by Robertson et al. (1995) which was designed to remediate the sustained attention deficits of a group of participants with right-hemisphere lesions. While participants performed a variety of routine tasks, the experimenter re-directed attention to the task by combining a loud noise with an instruction to attend, thus using intact bottom-up alerting pathways to re-orient attention. Participants were then gradually taught to initiate this alerting procedure using a self-generated verbal cue, finally learning to “self-alert” without needing to generate verbal cues at all. After training, all participants showed clinically significant improvements on a number of untrained behavioral tasks. SAT extends the behavioral training strategy with the addition of a biofeedback protocol. The objective of SAT is to gradually acquire the ability to control one’s alertness levels in a task-independent manner that can be potentially applied to a variety of real-life settings.

In recent years an increased number of studies have been conducted to evaluate the effects of EEG biofeedback, also known as “neurofeedback”, in the treatment of ADHD. Neurofeedback is a type of biofeedback that is aimed to teach or improve self-regulation over specific aspects of brain activity and implement these self-regulation skills in daily life (Arns et al., 2014). Similarly to SCR biofeedback used during SAT, during neurofeedback subjects receive on-line feedback on a particular brain wave and learn to self-regulate it. Recent reviews have found that neurofeedback can be successful in treating both hyperactive and inattentive symptoms in ADHD and that these positive effects lasted in time (Arns et al., 2014; Micoulaud-Franchi et al., 2014). However, neurofeedback has been criticized of methodological limitations and lack of an adequate control group (Arns et al., 2014). For example, one issue that complicates neurofeedback interventions is the fact that numerous training sessions are needed (30–40 sessions) to obtain significant improvements and therefore neurofeedback protocols may be complex to implement.

While the study by O’Connell et al. (2008) established the proof-of-concept of SAT with biofeedback in both adult controls and adults with ADHD, the training was limited to approximately 30 min and its impact on sustained attention was only probed in the immediate post-training interval. The present study implements a novel version of the SAT protocol that involves initial practice in the laboratory and then home-based training over an extended period of time (5-weeks). We compared performance of participants in the SAT group with performance of participants that were assigned to a Control Training (CT) condition. The aim of the CT procedure was to control for key non-specific elements of SAT, including interaction with the trainer, positive feedback and the placebo effect. Therefore, the control training group attended the same number of training sessions as the experimental group. We did not expect this CT to have a clinically meaningful effect on ADHD symptoms. This type of semi-active CT has been used in ADHD research before, for example in randomized controlled trials (RCTs) that examined the effects of neurofeedback (Arns et al., 2014). An important aspect related to the choice of the control paradigm was the face validity of this CT, as our participants were blind to their group condition. Our aim was to avoid participants’ complaints and to increase patients’ compliance with the program. The final decision was to combine attentional exercises with psycho-education regarding sustained attention.

The first aim of this study was to assess the effects of SAT on ADHD symptoms. The second aim was to investigate SAT impact on aspects of participants’ psychological functioning, as measured by participants’ self-efficacy ratings of their ability to cope with ADHD symptoms, and rates of psychiatric comorbidities that are often associated with ADHD (i.e., anxiety and depression). The third aim was to investigate whether SAT could improve untrained cognitive functions, such as selective and divided attention and executive functions that are not directed targeted by the intervention. The fourth aim was to investigate the impact of SAT on aspects of participants’ everyday life, as measured by participants’ subjective ratings on everyday life attention and memory problems. We hypothesizes that the SAT group will show more positive changes in ADHD symptoms, psychological functioning and untrained cognitive functions compared to the control group. We also expect greater improvements in subjective ratings of everyday life functioning in the SAT group in comparison with the control group.

## Material and methods

### Participants

Fifty-one participants were recruited from a specialist adult ADHD service, the Dean Clinic at St. Patrick’s Hospital, Dublin. Inclusion criteria were as follows: age between 18–50 years; full scale IQ > 80, assessed using Wechsler Adult Intelligence Scale—3rd Edition (WAIS–III, Wechsler, 1997); diagnosis of ADHD according to: DSM-IV criteria in both childhood and at present in adulthood using the Conners Adult ADHD Diagnostic Inventory for DSM-IV (CAADID; Epstein et al., 2000) and the Conners’ Adult ADHD Rating Scale (CAARS; Conners et al., 2003) and the Wender Utah Rating Scale (WURS), a retrospective measure of ADHD symptoms in childhood (Ward et al., 1993). The observer versions of both scales were also administered to a close family member or partner; self-reported clinically significant problems in daily life attributable to attentional, executive or arousal deficits (based on interview by a trained clinical psychologist); provision of informed consent. Exclusion criteria were: history of pervasive developmental disorders (e.g., Asperger’s syndrome, autism) or intellectual disability (IQ < 80); history or current diagnosis of epilepsy or other neurological condition (e.g., multiple sclerosis, motor neuron disease); history or current diagnosis of schizophrenia, bipolar disorder or other equivalently severe psychiatric condition; current primary diagnosis of substance misuse requiring treatment with priority (i.e., dependent on alcohol or illicit substances). However individuals with recreational alcohol and drug use were included as they are representative of the adult ADHD population.

Participants who met the inclusion criteria were randomly assigned using an automated minimization randomization procedure (Altman and Bland, 2005) which ensured that the two groups did not differ significantly in (a) gender; (b) prescribed psychotropic medication status; (c) alcohol and illegal drug use (sorted according to: alcohol consumption less than 35 units per week *and* no illegal drug more than once per month use vs. alcohol consumption more than 35 units per week *OR* illegal drug use more than once per month). Eight participants in the SAT group and six participants in the placebo group were taking psychostimulant medication for ADHD. Nine participants in the SAT group had comorbid disorders (one insomnia, one dyslexia and seven anxiety and depression) while four CT participants reported comorbid conditions (one depression and three anxiety and depression). The two groups did not differ in terms of gender, estimated IQ, measured by WAIS–III (Wechsler, 1997), mean years of education, and pre-training ADHD symptoms, as measured by the CAARS (Conners et al., 2003) and the WURS (Ward et al., 1993).

**Figure 1:**
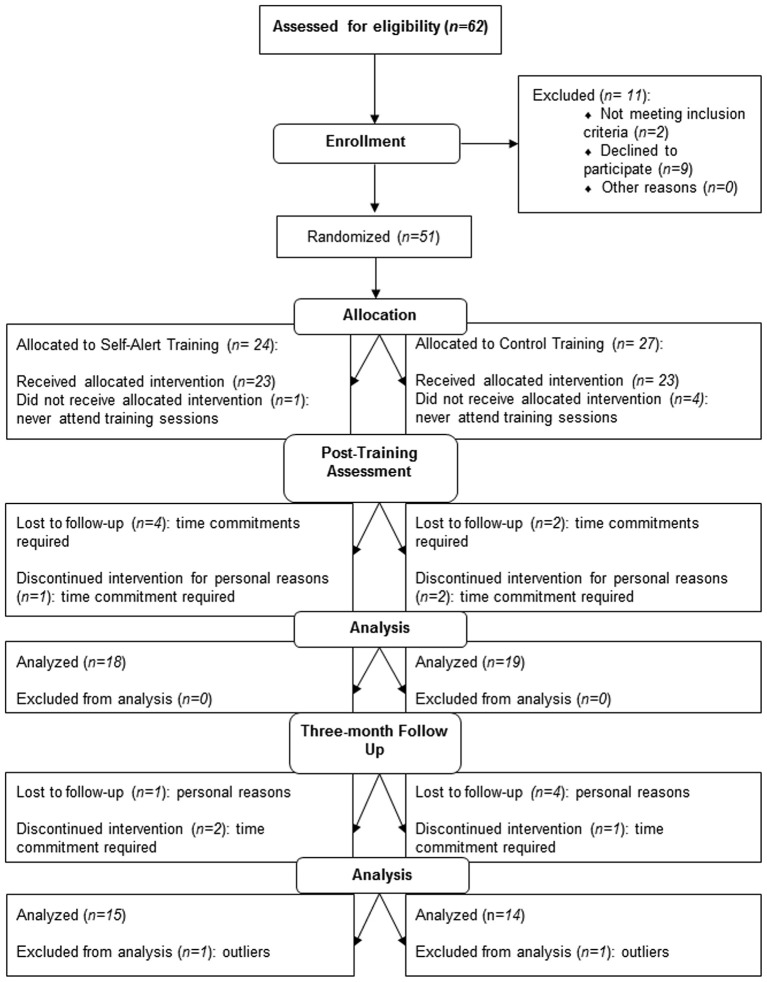
**Consolidated Standard for Reporting Trials (CONSORT) diagram showing the flow of participants through the trial and reasons for dropout**. SAT = Self-Alert Training; CT = Control Training.

Fourteen participants out or the 51 participants who were randomized dropped out during the 5-week training period leaving 18 participants in the SAT group and 19 in the control group who completed the training and pre and post-training assessment. The two groups did not differ in terms of age (*t*_(23)_ = 0.94, *p* = 0.35), years of education (*t*_(23)_ = 0.39, *p* = 0.70), estimated IQ (*t*_(23)_ = 1.56, *p* = 0.96). A further 8 participants dropped out of the study between the post-training and the three-month follow up assessment, leaving 15 participants in the SAT group and 14 participants in the CT group. Figure 1 showed the flow of participants through the study. The two groups did not differ for age (*t*_(16)_ = 0.99, *p* = 0.40), years of education (*t*_(16)_ = 1.22, *p* = 0.66), and estimated IQ (*t*_(16)_ = 1.33, *p* = 0.89). Demographic characteristics at each time point as well as CAARS and WURS scores at pre-training of SAT and CT participants are presented in Table 1.

**Table 1:** **Demographic characteristics at the three assessment points and measures of ADHD symptoms at pre-training in the SAT group (*n* = 24) and in the CT group (*n* = 27)**.

	Pre-training				Post-training		3-month follow-up
	SAT group^a^	CT group^a^	T_(49)_	p	SAT group	CT group	SAT group	CT group
N	24	27			18	19	15	14
Age	32.7 (12.4)	31.6 (11.3)	−0.40	0.69	32.8 (9.4)	31.4 (9.3)	32.6 (8.9)	31.5 (9.7)
Gender	16M; 8F	20M; 7F			12M; 7F	13M; 6F	9M; 6F	9M; 5F
Ethnicity: White	24	27			18	19	15	14
Years of education	15.9 (10.9)	14.7 (11.4)	0.39	0.70	15.7 (9.3)	14.3 (5.6)	15.9 (8.6)	14.7 (9.2)
IQ	112 (9.4)	109 (8.6)	0.94	0.36	110 (8.8)	109 (7.5)	112 (10.1)	108 (9.7)
Medications	8/24	6/27			7/18	6/19	5/15	5/14
Comordities	9/24	4/27			8/18	7/19	6/15	6/14
*Clinical symptoms of ADHD*
CAARS E- DSM IV inattention self	81.33 (9.38)	80.22 (13.20)	0.32	0.73
CAARS F- DSM IV hyperactivity self	65.92 (13.21)	66.00 (14.02)	0.02	0.98				
CAARS G- DSM IV total self	78.38 (9.05)	77.56 (14.73)	0.24	0.81
CAARS E- DSM IV inattention other	68.05 (10.64)	70.90 (11.63)	0.42	0.81
CAARS F- DSM IV hyperactivity other	63.80 (11.12)	64.55 (12.75)	−0.20	0.84
CAARS G- DSM IV total other	68.20 (10.61)	70.05 (12.09)	−0.52	0.61
WURS self	48.43 (19.33)	49.46 (29.45)	−0.08	0.93
WURS other	16.82 (6.54)	21.11 (7.70)	−0.95	0.10

*T-scores are reported for each variable. ^a^Values are mean (SD)*.

### Procedure

The study included three assessments (pre-training, post-training after 5-weeks of intervention, 3-month follow up), and 2 training sessions. Before starting the pre-training assessment, informed consent was obtained from each participant. During each assessment neuropsychological tests were carried out. A series of questionnaires were administered to participants and informant measures from a close relative or friend were also obtained. Participants’ randomization was conducted by a clinical psychologist based outside Trinity College Dublin using an automated procedure (Altman and Bland, 2005). ADHD participants were not aware of the type of their group allocation and researchers who conducted pre-training assessments as well as post-training and follow up assessments were blind to participants’ group allocation. Following the pre-training assessment, participants attended Trinity College Institute of Neuroscience for two training sessions, conducted by a trained research assistant. After the completion of both training sessions and assessments, participants were asked to practice the training themselves at home for 5-weeks. Participants in both groups were contacted once a week over the phone by a research assistant to assess their progress with their home-based training over the 5-week training period. All study procedures were approved by the Ethical Review Board of the School of Psychology, Trinity College Dublin in accordance with the Declaration of Helsinki.

### Measures

#### Adult ADHD symptoms

- The *Conners Adult ADHD Rating Scale: Long version* (CAARS-S:L; Conners et al., 2003).
- The *Conners Adult ADHD Rating Scale Observer: Long Version* (CAARS-O:L; Conners et al., 2003). To note, an insufficient number observer forms were returned to perform a well powered analysis (8 forms in the SAT group and 6 in the CT group); therefore these results are not described.

#### Psychological functioning

- The* Generalized Self Efficacy Scale* (GSES; Schwarzer and Jerusalem, 1995). The GSES is a 10-item scale designed to assess optimistic self-beliefs and copying skills. Participants rate each question on a scale ranging from 0 to 4, with higher scores indicating higher self-efficacy.
- The* Beck Anxiety Inventory* (BAI; Beck and Steer, 1993). The BAI consists of 21 questions about symptoms of anxiety. Participants rate each question on a scale from 0 to 3, with higher scores corresponding to greater anxiety.
- The* Beck Depression Inventory* (BDI-II; Beck et al., 1996). The BDI is a 21 questions inventory about the severity of depression’s symptoms Participants rate each question on a scale ranging from 0 to 3, with higher scores indicating higher depression.

#### Cognitive functions

- Two subtests from the* Test of Everyday Attention* (TEA; Robertson et al., 1994) were used: *Elevator Counting with Distraction*, in which participants had to listen to series of tones and count the high-pitched tones only. This task measures auditory selective attention. *Telephone Search While Counting* that involve counting a series of tones while looking for symbols on a telephone directory. This task measures divided attention and its final score is called: “Dual Task Decrement”.
- The* Hotel Task* (Manly et al., 2002) which measures executive functions and is designed to simulate typical day-to-day activities. The Hotel task is comprised of five distinct activities that would plausibly be completed in the course of running a hotel (i.e., checking guests’ bills, proofreading a leaflet on the hotel’s facilities, ordering labels with guests’ names in alphabetical order, sorting money, etc.,). The participants’ objective in this task is to try to complete as much as they can from each of the five activities over an allocated 10 min period. Performance in the Hotel Task is scored within two categories: Number of Attempted Tasks out of five, and time allocation, measured as the Total Deviation Time from an optimal time allocation of 2 min per activity.

#### Subjective measures

- The* Attention-Related Cognitive Errors Questionnaire (*ARCEQ; adapted from Cheyne et al., 2006). The ARCEQ is a 12 items scale that was used as a self-report measure of attention slips and absentmindness in everyday life. Participants rate the frequency with which they experience such slips in attention on a scale ranging from 1 to 5, with higher scores indicating higher absentmindness.
- The *Memory Failures Questionnaire* (EMFQ; adapted from Cheyne et al., 2006). It is structured in the same way as the ARCEQ and it is a self-report measure of minor memory failures that occur in everyday life. Participants rate the frequency with which they experience memory failures in a series of 12 items that are rated on a scale ranging from 1 to 5, with higher scores representing higher occurrence of memory failures.

### Self-Alert Training and Control Training (CT) protocols

Participants were asked to attend two training sessions. Each session was carried out on separate days and lasted on average 1 h and 20 min. Training sessions were a means of providing participants with psycho-education regarding sustained attention, arousal, and the role of noradrenaline in mediating levels of cognitive alertness. The role of the trainer was to facilitate and encourage participants’ development over the course of the training. Emphasis was thus placed on the role of the participant in what was essentially a “self-training” scheme.

#### Self-Alert Training (SAT)

**Figure 2:**
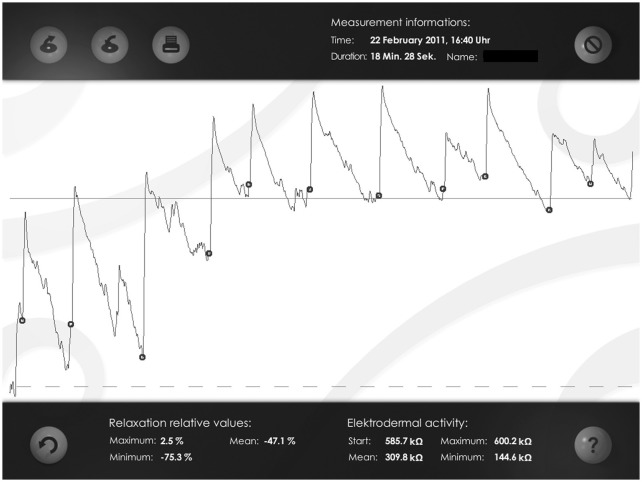
**Examples of participants’ biofeedback session with several successful alerts**. The dots indicate the start of a self-alert episode, which is followed by a clear increase (peak) in participants’ Skin Conductance Response (SCR).

SAT program consisted of an initial phase of psycho-education regarding the nature of alertness and attention, following which participants completed two questionnaires regarding their everyday life attention and memory difficulties. Following this, participants were taught to gain volitional control of their EDA using SAT in three main steps: (1) *Eliciting SCR by external alerting*. Participants were allowed to view the EDA readings on line and the meaning of this measurement was briefly explained. Then participants were presented with a loud alerting sound in order to demonstrate the responsiveness of their SCRs to changes in arousal. Participants were shown their SCR to this alert in real time. The experimenter asked participants to try to make a link between what they felt inside and the increase they saw in the line. This step was repeated five times and each time participants were allowed to view increases in their SCR waveforms on line (see Figure 2). Participants were instructed to relax as much as possible in between each alert to reduce the number of non-specific SCRs and thus ensure that increases in arousal were clearly observable in the EDA waveform; (2) *Cued internally generated SCRs*. The alerting sound was removed and the aim was for participants to begin producing internally driven increases in response to a verbal cue from the trainer (the word: “now”). Participants were asked to try to recreate the sudden increase in alertness they felt the first time the experimenter played the loud sound on the laptop. This step was repeated until participants could generate 5 clear increases in their SCR amplitudes. If participants had initial difficulties in generating SCR increases, the trainer guided them through the SAT technique step-by-step and invited them to try this again. There was no time limitation. To note, all our participants were able to learn the SAT technique by the end of the two training sessions. Participants were instructed to relax as much as possible in between each attempt in order to ensure that increases in arousal were readily observable; (3) *Self-initiated control over alertness*. In the final step, participants learned to take complete control of their EDA trace without any external prompt from the trainer. Participants were asked to say the word “now” when they were initiating a self-alert. This step was repeated with visual feedback until participants could generate 5 increases in SCR amplitudes. The same procedure was repeated but without visual feedback and participants were not able to view their EDA trace. Participants were asked to say the word “now” before initiating a self-alert and while the trainer checked their performance. This step was repeated until participants could generate 5 SCR increases. Finally, participants were instructed to save each biofeedback session on the laptop. Participants were instructed to relax as much as possible in between each attempt in order to ensure that increases in arousal were readily observable. Figure 2 shows examples of participants’ biofeedback session with several successful alerts.

A series of attentional exercises were also included in the program. To note, these exercises were included in the SAT program to provide participants additional occasions to practice the self-alert technique without the visual feedback from the biofeedback software. Participants were in fact asked to apply self-alerting strategies while practicing the attentional exercises in order to try and improve their performance. In this way they learned to control their alertness levels without visual feedback from their EDA trace, in a task-independent manner. The exercises consist of:

- *Sustained Attention to Response Task* (SART; Robertson et al., 1997*)*. Three versions of this task were included, each lasting 3 min and 10 s (126 trials). These were: Number SART, Auditory SART and Animal SART. In the Number SART, numbers from 1 to 9 were randomly presented in the center of participants’ training laptop and participants had to press the space bar for each number except 3. Similarly, in the Auditory SART, numbers from 1 to 9 were played on the laptop in a random order and participants had to listen to these numbers and press the space bar for every number except 3. In the Animal SART, nine different animal shapes were randomly presented in the center of the laptop’s screen and participants had to press the space bar for each animal shape except the kangaroo shape. At the end of each exercise participants received feedback on their performance in terms of number of correct responses, omission errors and commission errors.
- *Choice Reaction Time Task* (modeled from Logan et al., 1984). Two versions were included, each lasting 3 min and 45 s (150 trials). In the first version, an arrow pointing either to the left or to the right appeared in the center of the laptop’s screen. Participants had to press the corresponding arrow key on the laptop’s keyboard. In the second version, participants were presented with either the letter “O” or “X” that appeared in the center of the screen and they had to press the corresponding letter key on the laptop’s keyboard. Participants were instructed to answer as fast but also as accurately as possible. At the end of each exercise participants received feedback on their performance in terms of number of correct responses, omission errors and commission errors.
- *Listening Task* (this task was modeled from the Lottery task of the TEA, Robertson et al., 1994). Four different audio recordings were included consisting of recorded weather forecasts (downloaded from: www.rte.ie). The length of each recording was 5 min and 46 s. Participants had to listen carefully and indicate the number of times a particular word was pronounced (for example the word “wind” or “cloud”) by writing this number in a box that was presented on the laptop’s screen at the end of each exercise. Participants did not receive feedback on this task.

Each exercise was briefly demonstrated and participants were encouraged to apply the self-alert technique to improve their performance. Each practice of an attentional exercise was automatically saved by the training software in a database. Finally, participants were also invited to think about high-risk situations for attentional failures in their everyday life were encouraged to apply the SAT techniques in their day-to-day life during these problematic situations to increase their alertness. Participants were then provided with a laptop containing specialized SAT software and an EDA kit to take home. Participants were asked to practice 20 min of SAT-biofeedback exercises and 10 min of computerized exercises and to apply self-alerting during exercises for 5 days out of each week for 5-weeks.

#### Control Training (CT)

The CT session consisted of the same initial phase of psycho-education on sustained attention as well completion of two questionnaires on everyday life attention and memory difficulties, as in the SAT group. After this, the first type of attentional exercise (SART) was explained and participants were told that some studies had demonstrated that practicing certain types of attentional exercises had helped to improve sustained attention (all of this was designed to increase the face validity of the CT paradigm and to ensure participants’ blindness). The same attentional exercises were included in the CT program, as in the SAT program (SART, Choice Reaction Time Task and Listening Task). Participants were familiarized with each of the attentional exercises on the laptop provided. A brief discussion was conducted at the end of the last training session about participants’ experience of day-to-day life problematic situations and participants were invited to try to re-focus their attention during these situations. Participants in this group were asked to practice 20 min of computerized exercises for 5 days a week for 5-weeks. We believed that there was a risk of losing participants by asking them to practice more than 20 min, as the exercises were repetitive and any increase in training time requirements in this case have would likely led to participants’ frustration and loss of motivation to complete the training.

### Statistical analysis

Intention-to-treat (ITT) analysis was used. A mixed analysis of covariance (ANCOVA), including one between subjects variable, Group (two levels: SAT group, CT group), one within subjects variable, Time (three levels: pre-training assessment, post-training assessment, 3-month follow up) and one covariate, pre-training assessment was run for each measure. *Post hoc* comparisons were run using Bonferrorni correction to investigate between groups differences at each assessment session separately. Bonferroni correction accounted for all tests within each domain, as in our analysis it was applied for each outcome measure of ADHD symptoms, cognitive functions, psychological symptoms and everyday life subjective ratings. Table 2 presents the mean values and standard deviations obtained by the SAT group and the CT group at each time points for each measure and interactions effects of the ANCOVAs analysis for each measure.

**Table 2:** **Mean scores (and Standard Deviations) on ADHD symptom measures, social functioning and psychiatric comorbidities’ scales, neuropsychological tests and subjective attention and memory ratings for the SAT group and CT group, results of the ANCOVAs and between group effect sizes**.

	SAT group				CT group		
	Pre-training	Post-training	Follow-up	Pre-training	Post-training	Follow-up	Interaction
**1. Adult ADHD symptoms (CAARS Self-Report)**
Inattention and memory problems	72.39 (9.36)	63.67 (9.80)	71.81 (9.74)	72 (14.02)	69.79 (14.04)	66.33 (20.53)	*F* = 1.44, *p* = 0.24, *η*^2^ = 0.05
Hyperactivity	60.44 (11.66)	56.33 (11.86)	57.94 (10.03)	60.74 (9.97)	58.26 (11.26)	59.53 (12.39)	*F* = 0.13, *p* = 0.88, *η*^2^ = 0.01
Impulsivity and emotional lability	62.28 (12.02)	55.60 (11.57)	55.80 (12.68)	63.37 (15.66)	64.26 (14.89)	62.27 (14.52)	*F* = 4.06, *p* = 0.022*, *η*^2^ = 0.12
Problems with self-concept	59.83 (12.35)	53.72 (11.57)	51.00 (12.96)	60.47 (14.01)	60.89 (13.95)	61.13 (12.62)	*F* = 5.58, *p* = 0.007*, *η*^2^ = 0.16
DSM-IV inattentive symptoms	83.94 (7.33)	75.44 (9.15)	73.06 (14.60)	78.89 (14.68)	79.11 (11.21)	77.40 (14.04)	*F* = 3.96, *p* = 0.035*, *η*^2^.10
DSM-IV hyperactive symptoms	65.67 (14.53)	60.22 (12.96)	60.37 (13.37)	60.11 (15.46)	64.21 (15.24)	63.47 (15.62)	*F* = 0.79, *p* = 0.46, *η*^2^ = 0.03
DSM-IV total ADHD symptoms	80.00 (8.90)	71.33 (11.31)	71.00 (13.60)	75.79 (16.57)	75.53 (13.55)	73.87 (15.15)	*F* = 2.90, *p* = 0.13, *η*^2^.07
ADHD index	69.67 (7.63)	62.00 (10.04)	57.13 (12.44)	65.68 (13.56)	65.74 (13.14)	67.27 (12.01)	*F* = 7.81, *p* = 0.001*, *η*^2^.22
**2. Psychological functioning**
GSES	28.00 (5.70)	31.00 (4.76)	32.33 (5.42)	26.10 (5.65)	27.26 (6.70)	27.00 (6.77)	*F* = 4.55, *p* = 0.016*, *η*^2^.16
BDI	11.33 (8.20)	5.22 (5.27)	5.73 (5.33)	16.33 (12.20)	8.00 (8.26)	13.00 (9.03)	*F* = 3.81, *p* = 0.029*, *η*^2^.13
BAI	8.67 (6.18)	5.72 (5.53)	4.47 (4.94)	10.00 (7.41)	7.32 (6.48)	5.86 (4.43)	*F* = 0.99, *p* = 0.38, *η*^2^.04
**3. Cognitive functions**
Elevator with distraction (TEA)	7.50 (1.95)	9.11 (0.90)	8.75 (1.24)	7.11 (2.51)	7.21 (2.88)	7.80 (2.60)	*F* = 3.22, *p* = 0.038*, *η*^2^.13
Dual task decrement (TEA)	0.62 (0.84)	0.33 (0.61)	1.32 (4.01)	1.88 (3.26)	2.08 (1.99)	1.57 (1.86)	*F* = 0.74, *p* = 0.48, *η*^2^ = 0.03
Number of attempted tasks (Hotel task)	4.31 (0.79)	4.56 (1.03)	4.13 (1.20)	4.27 (0.80)	4.60 (0.91)	4.47 (0.83)	*F* = 0.96, *p* = 0.39, *η*^2^ = 0.03
Total deviation time (Hotel task)	165.68 (12.11)	63.61 (13.42)	83.02 (14.55)	145.54 (11.30)	125.12 (10.03)	110.03 (11.22)	*F* = 2.02, *p* = 0.12, *η*^2^ = 0.07
**4. Subjective measures**
ARCEQ	55.67 (8.17)	36.00 (8.18)	36.45 (9.35)	50.11 (12.63)	43.67 (10.97)	37.75 (11.09)	*F* = 7.16, *p* = 0.003*, *η*^2^ = 0.35
EMFQ	43.50 (12.04)	49.33 (10.39)	50.00 (7.99)	45.33 (11.62)	55.89 (11.32)	51.75 (13.64)	*F* = 0.47, *p* = 0.63, *η*^2^ = 0.03

Note. The degrees of freedom are 2,56 for the CAARS, 2,26 for GSES, 2.50 for BDI and BAI, 2,54 for neuropsychological tests and 2,26 for ARCEQ and EMFQ. Confident Interval is 95%. SAT = Self-Alert Training; CT = Control Training. *Indicated statistically significant difference.

## Results

### Adult ADHD symptoms (CAARS self report)

**Figure 3:**
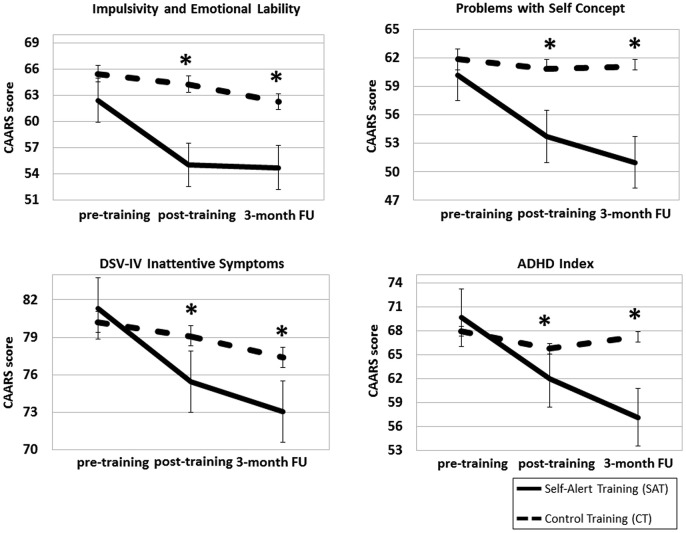
**Mean scores for the Self-Alert Training (SAT) and Control Training (CT) on the CAARS Self-Report at pre and post-training and at the 3-month follow up**. Participants in the SAT group showed significantly decreased ADHD symptoms compared to CT participants at post-training and after 3 months. Error bars represent standard errors. *Indicates statistically significant difference.

ANCOVA analysis showed that there was a significant Time × Group interaction for Impulsivity and Emotional Lability (*F*_(2,56)_ = 4.06, *p* = 0.022, *η*^2^ = 0.12). *Post hoc* comparisons indicated that the SAT group showed significantly lower scores compared to the CT group at the post-training assessment (mean difference = 3.69, SD = 1.63, *p* = 0.032) and that this effect was maintained at the 3-month follow up (mean difference = 4.24, SD = 1.58, *p* = 0.012). There was a significant Time × Group effect for Problems with Self Concept (*F*_(2,56)_ = 5.58, *p* = 0.007, *η*^2^ = 0.16), driven by significantly lower scores in the SAT group compared to the CT group at post-training (mean difference = 3.22, SD = 1.68, *p* = 0.041) and at the 3-month follow up (mean difference = 3.71, SD = 1.74, *p* = 0.032). There was a significant Time × Group effect for DSM-IV Inattentive Symptoms (*F*_(2,56)_ = 3.96, *p* = 0.035; *η*^2^ = 0.10). *Post hoc* analysis revealed that the SAT group had significantly lower scores compare to the CT group at post-training (mean difference = 4.18, SD = 1.37, *p* = 0.003) and that this effect was maintained at the 3-month follow up (mean difference = 5.64, SD = 2.02, *p* = 0.009). A significant Time × group interaction was found for ADHD Index (*F*_(2.56)_ = 7.81, *p* = 0.001; *η*^2^ = 0.22), driven by significantly lower scores in the SAT group compared to the CT group at post-training (mean difference = 4.44, SD = 1.45, *p* = 0.005) and at the 3-month follow up (mean difference = 4.86, SD = 1.82, *p* = 0.017). No significant Time × Group effects were found for Attention and Memory Problems, Hyperactivity and Restlessness, DSM-IV Hyperactive Symptoms and DSM-IV Total Symptoms (all *p* > 0.1). CAARS—Self Report scores at the three time points in the two groups are illustrated in Figure 3.

### Psychological functioning

**Figure 4:**
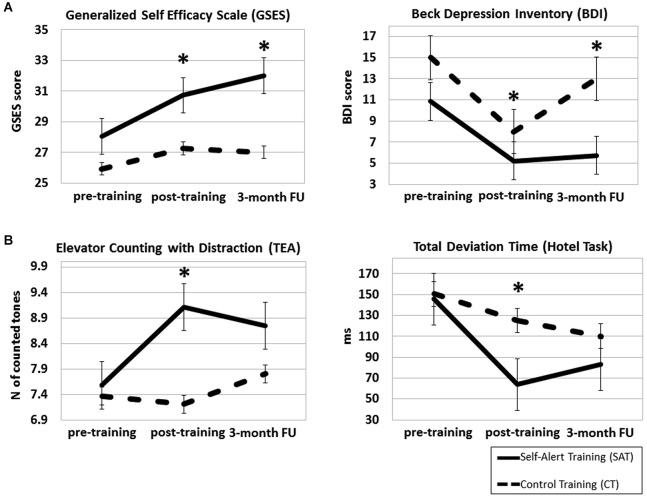
**Mean scores for the Self-Alert Training (SAT) and the Control Training (CT) group on the Generalized Self-Efficacy Scale (GSES) and Beck Depression Inventory (BDI) (A) and on the Elevator Counting with Distraction and Total Deviation Time (B) at pre and post-training and at the 3-month follow up**. The SAT group showed increased significantly self-efficacy scores, decreased depressive symptoms at both assessments and improved scores in both cognitive tasks after training. Error bars represent standard errors. *Indicates statistically significant difference.

ANCOVA analysis showed a significant Time × Group interaction for the GSES (*F*_(2,26)_ = 4.55, *p* = 0.016, *η*^2^ = 0.16). *Post hoc* comparisons indicated that the SAT group showed significantly higher self-efficacy scores compared to the CT group immediately after training (mean difference = −2.90, SD = 0.77, *p* = 0.001) and at the 3-month follow up (mean difference = −4.02, SD = 1.02, *p* = 0.001). A significant Time × Group effect emerged for The BDI (*F*_(2,50)_ = 3.81, *p* = 0.029, *η*^2^ = 0.13), driven by significantly lower depression ratings in the SAT group than in the CT group at post-training (mean difference = 7.81, SD = 1.23, *p* < 0.000) and at the 3-month follow up (mean difference = 4.82, SD = 1.26, *p* = 0.001). No significant group difference emerged for the BAI (*p* = 0.38). GSES and BDI scores at the three assessment points for the SAT group and CT group are shown in Figure 4A.

### Cognitive functions

ANCOVA analyses revealed a significant Time × Group effect on the Elevator Counting with Distraction (*F*_(2,54)_ = 3.22, *p* = 0.038, *p* = 0.013, *η*^2^ = 0.12). *Post hoc* comparisons indicated that the SAT group improved significantly more than the CT group at post-training (mean difference = −0.85, SD = 0.35, *p* = 0.023), but this improvement was not maintained at the 3-month follow up (mean difference = −0.17, SD = 0.23, *p* = 0.47). The ANCOVA for the Total Deviation Time in the Hotel Task was not significant (*F*_(2,54)_ = 1.02, *p* = 0.14, *p* = 0.013, *η*^2^ = 0.07), however *post hoc* analysis revealed that the SAT group improved significantly more than the CT group at the post-training assessment (mean difference = 44.69, SD = 19.08, *p* = 0.027), but this improvement was not maintained at the 3-month follow up (mean difference = 21.65, SD = 13.55, *p* = 0.06). No significant differences between groups emerged on the Dual Task Decrement and on Number of Attempted Tasks in the Hotel Task (both *p* > 0.2). Figure 4B showed the two groups’ scores on cognitive tasks at the three time points.

### Subjective measures

**Figure 5:**
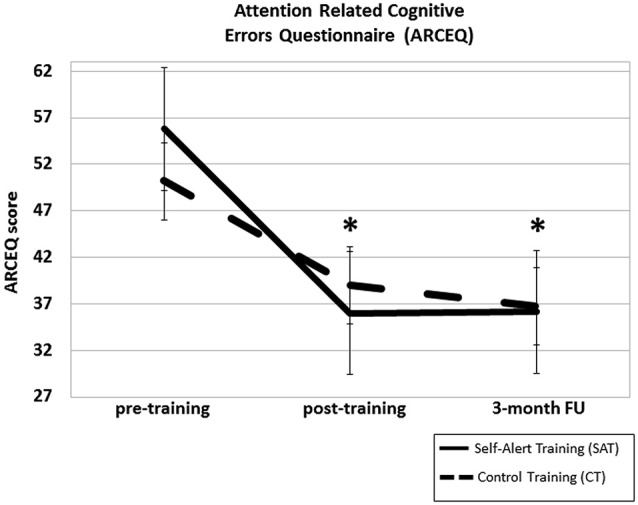
**Mean scores for the Self-Alert Training (SAT) and Control Training (CT) group at pre and post-training and at the 3-month follow up on the Attention related Cognitive Error Questionnaire (ARCEQ)**. SAT participants significantly decreased their subjective ratings of attentional slips compared to CT participants after training and at the 3-month follow up. Error bars represent standard errors. *Indicates statistically significant difference.

ANCOVA analysis showed a significant Time × Group interaction for the ARCEQ (*F*_(2,26)_ = 7.16, *p* = 0.003, *η*^2^ = 0.35). *Post hoc* comparisons indicated that the SAT group showed significantly lower ratings of attentional slips compared to the CT group at post-training (mean difference = 10.35, SD = 1.62, *p* < 0.000) and that this effect was maintained at the 3-month follow up (mean difference = 12.78, SD = 1.70, *p* < 0.000). No significant difference between groups emerged for the MFQ (*p* = 0.63). ARCEQ scores in the two groups at the three assessment points are shown in Figure 5.

### Individual differences in time spent training

Participants in the SAT group spent a mean of 2.7 h in attentional exercises (range: 9.40 h to 10.5 min) and they completed, on average, 49.1 attentional exercises (range: 168–3; SD = 48.5) over the 5-weeks. SAT participants did a mean of 36.6 biofeedback sessions (range: 116–4) and the mean time spent in biofeedback training was 3.5 h (range: 21.36 h-38 min) over the 5 week period. The mean duration of a biofeedback session was 9.2 min (range: 17.59–3.54 min; SD = 6.7). Participants in the CT group spent a mean of 4.2 h in attentional exercises (range: 11.54 h-10.5 min) and they completed, on average, 57.5 attentional exercises (range: 204–3; SD = 68.8) over 5-weeks. Independent sample *t*-tests showed that there was no significant difference between the SAT group and the CT group in the mean number of attentional exercises practiced (*t*_(29)_ = −1.076, *p* = 0.291) and in the mean total training time (*t*_(31)_ = 1.554, *p* = 0.130).

**Figure 6:**
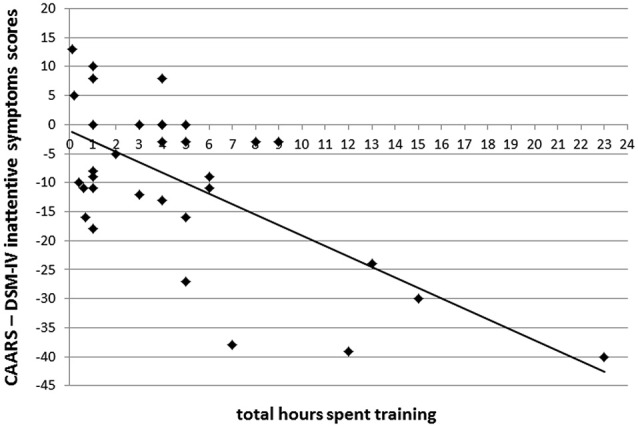
**Scatter plot depicting the relationship between the total number of hours spent training and proportional reduction in DSM-IV Inattentive Symptoms scores for SAT participants**.

In order to examine whether there was a dose-response relationship between time spent training and the degree of improvement, correlations were run between the total amount of time sent training by SAT participants and the proportional improvement at post-training assessment for all measures that showed a significant Time × Group interaction. Proportional improvement was calculated by subtracting post-training scores from pre-training scores. There was a significant negative correlation between total time spent training and participants’ ratings on CAARS inattentive symptoms (*r*_(48)_ = −0.77, *p* < 0.000), indicating that longer SAT practice was associated with greater reductions in ADHD inattentive symptoms. There were no other significant correlations (all *p* > 0.1). Figure 6 illustrates a scatter plot showing the significant correlation between ADHD inattentive symptoms and time spent training.

## Discussion

The first aim of this study was to evaluate the efficacy of partially home-based SAT program on ADHD symptoms in adults with ADHD. The results of the study showed that adults with ADHD who received SAT exhibited significantly reduced ADHD inattentive and impulsive symptoms, as measured by the CAARS—Self Report questionnaire both immediately post-training and at the three-month follow up, while adults with ADHD allocated to the CT group showed no significant changes in ADHD symptoms at either of the two time points. Furthermore, a significant correlation emerged indicating that participants who showed greater reduction on ADHD inattentive symptoms were also those who spent more time in training. This may suggest that SAT beneficial effects on inattention may be modulated by the amount of time and effort dedicated to practising the technique. The second aim of this study was to evaluate the impact of SAT on participants’ psychological functioning. The SAT group showed significant improved self-efficacy ratings indicating that SAT can instil confidence in participants’ ability to control their symptoms. Reduced depressive symptoms were also found in the SAT group. It may be that SAT participants’ improved sense of self-efficacy over their ADHD symptoms may also have had the effect of reducing depressive symptoms and negative attitude. This result is also consistent with previous research demonstrating that cognitive training can improve depressive symptoms and dysfunctional beliefs in patients with depression (Trebo et al., 2007; Wolinsky et al., 2009). The third aim of this study was to investigate SAT effects on a range of untrained cognitive functions. Improvements were observed at post-training in the SAT group on selective attention and executive functions. It is important to note that the training protocol did not involve practising these neuropsychological tasks and, as a result, these behavioral effects may indicate that SAT can lead to a generalization of training effects to untrained cognitive tasks. The improvements on inattentive symptoms following SAT may also have led to a generally enhanced level of attention and this can have facilitated SAT participants’ performance on these untrained tasks. However, these effects were not maintained at the three-month follow up, thus suggesting that SAT needs to be implemented for a longer period to maintain these improvements. Clearly, replications of these findings are needed to better explain these transfer effects. The fourth aim of this study was to assess the impact of SAT on aspects of participants’ everyday life functioning. SAT participants showed significant reduction of everyday life attentional slips, as measured by subjective ratings in the ARCEQ. This suggests that ADHD participants have learned to successfully apply the training strategies in a range of real life situations.

Our results indicate that the SAT program may lead to reduced self-reported ADHD symptoms and that these effects lasted 3-months after the end of training. Similarly to our results, previous studies using behavioral treatments as well as neurofeedback interventions have shown significant improvements in both ADHD inattentive and hyperactive symptoms and that these positive effects lasted in time, as measured by distal follow up assessments (Stevenson et al., 2003; Safren, 2006; Arns et al., 2014). Our results also suggest that SAT may result in improved self-efficacy and depressive symptoms. Reductions in depressive symptoms as well in anxiety have been found in previous research using CBT and psychoeducation (Solanto et al., 2008; Vidal et al., 2013), while, to our knowledge, no previous studies of neurofeedback interventions in adults with ADHD have reported increases in these psychiatric comorbidities (Simkin et al., 2014). Improved performance on untrained cognitive tasks at post-training emerged in this study. A potential limitation of previous studies using behavioral interventions in adult ADHD is that while these approaches have proven efficacy in reducing behavioral problems, such as disruptive behaviors, they generally do not target the underlying neuropsychological deficits in ADHD. Neuropsychological functions such as sustained attention and executive functions are vital in adults with ADHD for continuing learning and for academic success (Dupaul, 2006). Very few studies have investigated generalization effects of neurofeedback on neuropsychological functions in ADHD and these have shown inconclusive results. For example, a recent study (Bink et al., 2014) found no improvements after neurofeedback in executive functions in a group of adolescents with ADHD while another study (Steiner et al., 2014) reported moderate improvements in an index of attention and executive functions in children with ADHD. This evidence suggests that more research is needed to develop behavioral treatments that can address underlying neuropsychological deficits in adults with ADHD. Our results also found reduced subject ratings of attentional slips. To note, the final aim of SAT was to teach participants the ability to self-alert in real life settings to flexibly increase attention, thus emphasis was placed on the application of self-alert techniques in real life. This result indicates that SAT can potentially be used to help adults with ADHD to manage and control their own attentional symptoms in day-to-day settings. To our knowledge, no neurofeedback studies have implemented techniques to promote generalization to daily life (Arns et al., 2014). Research on psychoeducation in adults with ADHD (Vidal et al., 2013) has found improvements in participants’ quality of life after treatment. Clearly, new strategies in behavioral interventions should be developed that can address everyday life functional impairments in adults with ADHD in order to increase their quality of life.

Some methodological advantages also emerged in our study. For example, compared to classic neurofeedback protocols, that require at least 30 training sessions, or other more complex behavioral interventions, our training program may be easier to implement as it requires participants to attend two lab-based training sessions while the remaining part of the program is self-administered and can be carried out at participants’ homes. This may increase treatment’s flexibility and may provide a more user-friendly approach for participants. Furthermore, studies that have evaluated the efficacy of behavioral intervention as well neurofeedback protocols in ADHD (Sonuga-Barke et al., 2013; Micoulaud-Franchi et al., 2014) have highlighted a common issue regarding assessments’ blindness to evaluate efficacy of intervention. In fact the majority of behavioral treatment studies in ADHD have employed ratings completed by assessors who were aware of the participant’s treatment conditions (Sonuga-Barke et al., 2013). Recent reviews on neurofeedback trials have also stressed the importance of blind assessments (Sonuga-Barke et al., 2013; Micoulaud-Franchi et al., 2014), as results indicated that probably blinded assessment is influenced more by random error and is more unstable than unblinded assessment (Micoulaud-Franchi et al., 2014). In our study, participants’ and observers were both blind to their group allocation and efforts were made to maintain blindness of participants, observers and researchers during the study.

However, several limitations to this study have to be mentioned. First, it was not possible to examine the results of the CAARS-Observer Forms, as an insufficient number of these forms were received at both post-training assessments for a meaningful analysis. In future research, more efforts should be made to explain to participants the importance of returning observer forms in order to obtain corroborative measures of changes in ADHD symptoms. Multi-informant measures would be important to confirm the current results in self-reported ADHD symptoms. Second, a moderately high number of participants dropped out of the study (9 SAT participants and 13 CT participants) and this reduces the impact of our findings. To note, in order to reduce drop-outs, weekly phone calls and daily text messages were sent to each participant during the training period to check on their practice and to reinforce compliance. Despite these efforts, a number of participants still dropped out of the study and, during weekly phone calls, these participants revealed that the time commitment required from the training program was too high. One solution to avoid future drop-outs may be to conduct a more detailed investigation of participants’ daily commitments (i.e., work and family commitments) at recruitment stage and to discuss with participants the practical aspects of their daily training and how to embed this into their day-to-day schedules. This may help to increase participants’ awareness on the training’s commitments and feasibility. Another suggestion for future studies may be to involve participants’ partner or a close relative into the training program to increase treatment’s compliance and persistence. Third, the control group was required to simply practise repetitive attentional exercises. This likely resulted in a lower level of engagement in the control participants, as suggested by higher drop-out rates in the CT group compared to the SAT group. More engaging CT program is desirable in order to increase control participants’ motivation to continue their training. Fourth, we used subjective questionnaires’ ratings to evaluate the impact of SAT in everyday life. However, measures of everyday life functioning are available that have higher ecological validity, such as the Goal Attainment Scaling (GAS; Kiresuk et al., 2009). This scale involves the selection of participants’ tailored everyday life goals based on aspects of daily functioning participants would like to improve, and related subjective goal ratings to evaluate pre-post training changes (Clare et al., 2009). The use of a goal attainment procedure would enable to better assess the training’s ecological validity.

Despite these difficulties and given the limitations of classic pharmacological and behavioral treatments for ADHD (Asherson, 2005; Sonuga-Barke et al., 2013), it is hoped that this type of behavioral training may in future be used to form the basis for a clinically oriented method in the multimodal treatment of ADHD in adults. Future work should aim to conduct a full scale RCT to allow to investigate consistent effects of SAT in a larger sample of adults with ADHD. Given the life-long nature of ADHD (Faraone et al., 2006), future research should also include several distal follow-up assessments to explore long-term effects of SAT in adult ADHD.

## Authors and contributors

Design of the work, data acquisition, analysis and interpretation: Simona Salomone (design, data collection, analysis and interpretation), Grainne Ruth Fleming (data collection), Jacqueline Marie Shanahan (data collection), Marco Castorina (programming of the training programs and data collection), Redmond G. O’Connell (design, and data interpretation), Jessica Bramham (design and data interpretation), Ian H. Robertson (design and data interpretation). Drafting the work or revising it critically for important intellectual content: Simona Salomone, Grainne Ruth Fleming, Redmond G. O’Connell, Jessica Bramham, and Ian H. Robertson. Substantial contributions to the conception or design of the work: Simona Salomone, Redmond G. O’Connell, Jessica Bramham, Ian H. Robertson. Final approval of the version to be published: Simona Salomone, Grainne Ruth Fleming, Jacqueline Marie Shanahan, Marco Castorina, Redmond G. O’Connell, Jessica Bramham, Ian H. Robertson. Agreement to be accountable for all aspects of the work in ensuring that questions related to the accuracy or integrity of any part of the work are appropriately investigated and resolved: Simona Salomone, Grainne Ruth Fleming, Jacqueline Marie Shanahan, Marco Castorina, Redmond G. O’Connell, Jessica Bramham, and Ian H. Robertson.

## Conflict of interest statement

The authors declare that the research was conducted in the absence of any commercial or financial relationships that could be construed as a potential conflict of interest.
